# Troponin Variants as Markers of Skeletal Muscle Health and Diseases

**DOI:** 10.3389/fphys.2021.747214

**Published:** 2021-09-27

**Authors:** Monica Rasmussen, Jian-Ping Jin

**Affiliations:** ^1^ Department of Physiology, Wayne State University School of Medicine, Detroit, MI, United States; ^2^ Department of Physiology and Biophysics, University of Illinois at Chicago, Chicago, IL, United States

**Keywords:** skeletal muscle, troponin I, troponin T, isoform, splice form, development, adaptation, myopathy

## Abstract

Ca^2^^+^-regulated contractility is a key determinant of the quality of muscles. The sarcomeric myofilament proteins are essential players in the contraction of striated muscles. The troponin complex in the actin thin filaments plays a central role in the Ca^2+^-regulation of muscle contraction and relaxation. Among the three subunits of troponin, the Ca^2+^-binding subunit troponin C (TnC) is a member of the calmodulin super family whereas troponin I (TnI, the inhibitory subunit) and troponin T (TnT, the tropomyosin-binding and thin filament anchoring subunit) are striated muscle-specific regulatory proteins. Muscle type-specific isoforms of troponin subunits are expressed in fast and slow twitch fibers and are regulated during development and aging, and in adaptation to exercise or disuse. TnT also evolved with various alternative splice forms as an added capacity of muscle functional diversity. Mutations of troponin subunits cause myopathies. Owing to their physiological and pathological importance, troponin variants can be used as specific markers to define muscle quality. In this focused review, we will explore the use of troponin variants as markers for the fiber contents, developmental and differentiation states, contractile functions, and physiological or pathophysiological adaptations of skeletal muscle. As protein structure defines function, profile of troponin variants illustrates how changes at the myofilament level confer functional qualities at the fiber level. Moreover, understanding of the role of troponin modifications and mutants in determining muscle contractility in age-related decline of muscle function and in myopathies informs an approach to improve human health.

## Introduction

The sarcomere is the functional unit of striated muscles, and the sarcomeric myofilament proteins are key players in muscle functions. A sarcomere of vertebrate skeletal muscle contains myosin thick filaments, actin thin filaments, and titin and nebulin filaments, as well as accessary proteins such as myosin binding protein C and the troponin-tropomyosin (Tm) regulatory complex. Skeletal muscle contraction is initiated by an influx of Ca^2+^ or physical interactions of the dihydropyridine receptor with the ryanodine receptor Ca^2+^ ion channel, mediating the release of more Ca^2+^ from the sarcoplasmic reticulum, and Ca^2+^ binding to troponin will induce a series of conformational changes in the myofilaments to activate myosin ATPase and myosin-actin cross-bridge cycling, initiating power strokes that shorten the sarcomere (Gordon et al., 2000; Petegem, 2012).

Vertebrate skeletal muscles are categorized as fast-twitch anaerobic and slow-twitch aerobic muscles by fiber type contents, e.g., type 1 (slow), type 2A (oxidative fast), and type 2B (glycolytic fast) fibers in humans (Schiaffino and Reggiani, 2011). Based on types of myosin isoenzymes with different ATPase activity, muscle fiber types are further delineated into type 1, type 2A, type 2B, and type 2X (Schiaffino, 2018), where muscles may contain “pure” fibers expressing a single myosin heavy chain (MHC) isoform and, more frequently, “hybrid” fibers express multiple MHC isoforms (Pette and Staron, 2000). Humans express three MHC isoforms in adult skeletal muscle, MHC I, MHC IIa, and MHC IIx/d encoded by *MYH7*, *MYH2*, and *MYH1* genes, respectively, as well as embryonic and neonatal isoforms encoded by *MYH3* and *MYH8* genes (Galler et al., 1997; Schiaffino and Reggiani, 2011). Commonly studied small mammals such as mice, rats, and rabbits express a fourth isoform MHC IIb encoded by the *Myh4* gene (Scott et al., 2001). Heterogenic fibers are commonly found in mammalian skeletal muscles (Talbot and Maves, 2016).

Muscles playing a role in body posture are composed of more slow type I fatigue resistant fibers whereas muscles important for movement are composed of a higher percentage of fast type II fibers. Many muscles perform both roles and are heterogenic with regard to fiber type (Johnson et al., 1973). Muscles that perform specialized functions can have a rather pure fiber content, such as the tongue and esophageal muscles which have almost entirely fast fibers (Prigozy et al., 1997; Zhao and Dhoot, 2000) whereas the masticatory and extraocular muscles are mixed fiber types and show significant variability between individuals (Wasicky et al., 2000; Rowlerson et al., 2005; Bicer et al., 2011). Significant heterogeneity exists when classifying muscle fibers based on myosin composition (Salviati et al., 1982) and MHC isoform alone is not sufficient to fully characterize a muscle fiber (Terry et al., 2018).

**Figure 1:**
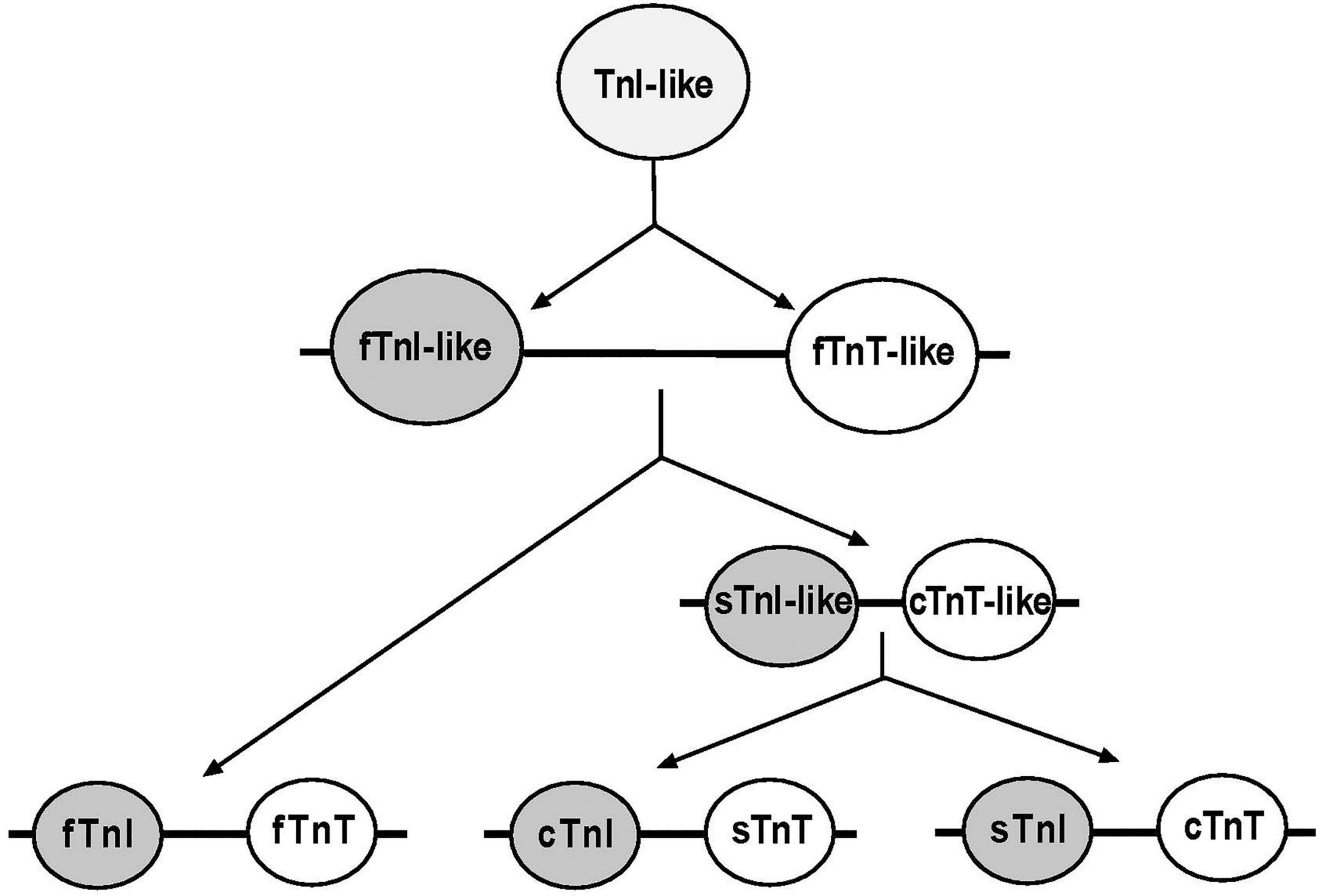
Evolutionary lineage of TnI and TnT isoforms. Phylogenetic studies revealed that TnI and TnT genes were likely emerged from a TnI-like ancestor *via* gene duplication. The first linked gene pair was fast TnI (fTnI)-like and fast TnT (fTnT)-like. Subsequent gene duplication events during vertebrate evolution added a slow TnI (sTnI)-like and cardiac TnT (cTnT)-like pair that further duplicated to add the cardiac TnI (cTnI) and slow TnT (sTnT) pair of genes (Chong and Jin, 2009).

Ca^2+^-regulation is essential for muscle contraction and relaxation (Metzger and Moss, 1990). Troponin is the key calcium-dependent regulator of striated muscles (Greaser and Gergely, 1971). The troponin complex is a heterotrimer composed of troponin C (TnC), the Ca^2+^-binding subunit, troponin I (TnI), the inhibitory subunit, and troponin T (TnT), the Tm-binding subunit. The troponin subunits are encoded by separate genes that have evolved into muscle fiber-type-specific isoforms. Whereas TnC has only two isoforms, one in fast skeletal muscle and the other in cardiac and slow skeletal muscles, TnI and TnT were evolved from a TnI-like ancestor and each has three isoforms for cardiac, slow skeletal, and fast skeletal muscles (Figure 1; Chong and Jin, 2009; Sheng and Jin, 2014). Polyploid vertebrate skeletal muscle cells can express a single class of troponin isoforms in a highly fiber-type-specific manner with coupled expression of slow TnI and slow TnT in pure slow fibers and fast TnI and fast TnT in pure fast fibers (Brotto et al., 2006).

Owing to troponin’s central role in controlling the Ca^2+^-handing of striated muscles, troponin variants can be used as muscle-specific markers to assess muscle fiber type and quality. In this focused review, we will address the regulation and function of troponin isoforms and alternative splice forms in defining skeletal muscle fiber type, developmental and differentiation states, contractile function, age-related decline, and other physiological and pathophysiological adaptations.

## Expression of Troponin Isoforms is Fiber-Type Specific with Functional Significance

Troponin emerged ~700 million year ago when animals evolved coordinated movement (Cao and Jin, 2020). Complex divergence of troponin subunit proteins has evolved in vertebrates with functional impacts (Jin et al., 2008; Sheng and Jin, 2014). Data from half century of research on the structure–function relationships of troponin subunits provide informative insights into the significance of troponin isoform expression in muscle health and diseases.

### Troponin C

**Figure 2:**
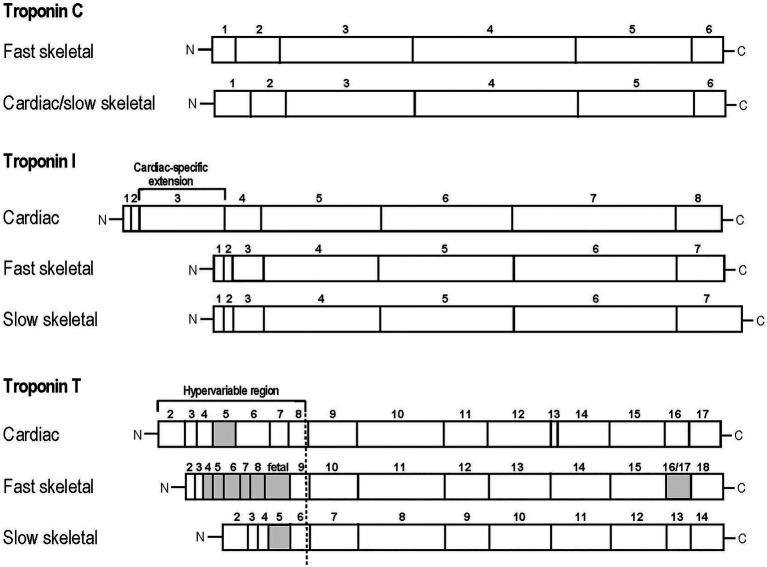
Linear protein maps of troponin subunit isoforms. Linear structures of the two TnC, three TnI and three TnT isoforms are shown with segments encoded by individual exons outlined. The adult heart-specific N-terminal extension of cardiac TnI and the alterative splicing-generated variable regions of TnT are indicated. The alternatively spliced exons are labeled with the gray boxes.

TnC is a member of the calmodulin super family of Ca^2+^ receptor proteins (Wilkinson, 1980; Li and Hwang, 2015). Vertebrate TnC has two isoforms encoded by homologous genes in slow skeletal/cardiac (*TNNC1*) and fast skeletal (*TNNC2*) muscles (Figure 2; Li and Hwang, 2015). Binding of Ca^2+^ to the N-terminal domain of TnC induces changes in troponin conformation and the position of Tm on the actin filament to allow myosin head binding to actin and the initiation of contraction. Fast TnC has two regulatory Ca^2+^ binding sites whereas slow/cardiac TnC has only one (Van Eerd and Takahashi, 1976; Putkey et al., 1991). While Ca^2+^ activations of fast skeletal and cardiac muscles show differences in length-tension relationship, evidence suggests that the TnC isoforms are not a determining factor, consistent with the notion that TnC is merely a Ca^2+^-sensing switch that relays Ca^2+^ signal to the other subunits of troponin, which then function to modulate length–tension relationship (Moss et al., 1991; Wang and Fuchs, 1994; McDonald et al., 1995). Nonetheless, single fiber studies show that fibers that contain a pure MHC I or MHC II isoform contain the corresponding slow or fast TnC isoform, whereas hybrid slow and fast myosin fibers contain both slow and fast isoforms of TnC (O’Connell et al., 2004), rendering TnC isoform as a muscle fiber-type-specific marker.

### Troponin I

TnI is the inhibitory subunit of troponin and functions to inhibit actomyosin ATPase, critical for muscle relaxation when cytosolic Ca^2+^ decreases. In vertebrates, TnI is encoded by three distinct isoform genes that are specifically expressed in slow skeletal (*TNNI1*), fast skeletal (*TNNI2*), and cardiac (*TNNI3*) muscles (Hastings, 1997; Mullen and Barton, 2000). The C-terminal and middle regions of the TnI isoforms are conserved while cardiac TnI possesses a unique N-terminal extension that is not present in the skeletal muscle isoforms (Figure 2; Sheng and Jin, 2016).

Fast skeletal muscle TnI is expressed in fast-twitch muscles like the extensor digitorum longus and psoas (Hettige et al., 2020) corresponding to lower Ca^2+^ sensitivity than that of slow TnI, suggesting the fast skeletal muscle is in a lower state of activation compared to cardiac and slow skeletal muscle sarcomeres (de Tombe et al., 2007).

While cardiac TnI is uniquely expressed in the adult vertebrate heart, embryonic heart expresses solely slow skeletal muscle TnI that confers higher Ca^2+^-sensitivity and resistance to acidic pH, a condition that pre-supposes its resistance to exercise-induced physiological stress and acidosis in slow skeletal muscle (Westfall et al., 1997). The switch to cardiac TnI occurs in developing human hearts around 20days after birth correlating with the cease of hypoxia and acidosis in fetal heart (Hunkeler et al., 1991). The exchangeability between cardiac and slow skeletal muscle TnI with functional impacts is consistent with the notion that TnI isoform regulation impacts muscle contractility.

### Troponin T

TnT is the Tm-binding subunit of troponin. It functions to anchor the troponin complex onto the thin filament as well as transduce calcium-dependent conformational changes in TnC to configure the actin thin filament and regulate muscle contraction and relaxation (Wei and Jin, 2011). Three genes in vertebrates code for three fiber-type-specific TnT genes: *TNNT1* encodes slow skeletal muscle TnT, *TNNT2* encodes cardiac muscle TnT, and *TNNT3* encodes fast skeletal muscle TnT (Wei and Jin, 2016). Whereas the N-terminal region shows significant diversity between isoforms, the middle and C-terminal regions of TnT are highly conserved (Jin et al., 2008). Alternative splicing of multiple exons further adds to the diversity of TnT structure and function (Figure 2; Wei and Jin, 2016).

Fast skeletal muscle TnT is specific to fast twitch fibers, where it undergoes complex N-terminal alternative splicing to produce a high to low molecular weight switch during post-natal development (Wei and Jin, 2016). This switch imparts a transition from low to high isoelectric point splice forms as the result of alternative inclusion of exons encoding acidic amino acid-rich N-terminal segments (Wang and Jin, 1998). Cardiac TnT also undergoes developmental alternate splicing that generates a switch from acidic embryonic to basic adult splice forms (Jin and Lin, 1989) with the embryonic form corresponding to higher myofilament calcium sensitivity (Gomes et al., 2004). Skeletal muscle fibers have higher cooperativity than cardiac muscle fibers, and exchanging fast skeletal muscle TnT for cardiac TnT in adult transgenic mouse hearts results in an increase in cooperativity of Ca^2+^ activation of force (Huang et al., 1999a), highlighting the functional significance of fiber-type-specific TnT expressions. Slow skeletal muscle TnT is expressed in slow twitch fibers, where N-terminal alternative splicing produces high and low molecular weight variants (Jin et al., 1998a; Krishan et al., 2000). The two high molecular weight splice forms show high expression in rat soleus muscle, while the two low molecular weight splice forms show nominal expression in fast extensor digitorum longus and tibialis anterior muscles. Studies exchanging fast TnT with slow TnT in skinned fibers resulted in an increase in sensitivity to Ca^2+^-activation but a decrease in cooperativity, consistent with slow fibers being more sensitive to calcium than fast fibers (Kischel et al., 2005).

Concurrent with the developmental exclusion of N-terminal embryonic exons, a post-hatching inclusion of alternatively spliced exons adds a unique Glu-rich metal binding segment in the N-terminal region of adult avian pectoral muscle fast TnT which functions in modulating molecular conformation and affinity for Tm as well as Ca^2+^ sensitivity of muscle contraction (Ogut and Jin, 1996; Ogut et al., 1999), potentially important for flight activities (Cao and Jin, 2020).

## Expression of Troponin Isoforms are Regulated During Development and Myogenesis

### Myogenesis

Troponin subunits are striated muscle-specific proteins with significant isoform and splice form regulation during myogenesis. Vertebrate skeletal muscle myogenesis begins with stem cells differentiating into primary myoblasts that develop into primary myofibers in neonates, which form the scaffold upon which secondary myoblasts ultimately form secondary myofibers in adult vertebrates (Chal and Pourquié, 2017). Evidence indicates that primary myoblasts express primarily slow-type myofilament proteins and secondary myoblasts express primarily fast-type myofilament proteins, though isoform switching among a number of myofilament proteins in developing fibers makes this assessment more complex (Condon et al., 1990; Hallauer and Hastings, 2002a). Still, such isoform switching demonstrates that primary and secondary myoblasts are not confined to a specific fate as slow or fast fibers (Guerrero et al., 2014).

It has been suggested that differentiating primary and secondary myoblasts show a common progenitor profile of troponin isoforms regardless of fiber type or myosin isoforms, and that *de novo* assembly of sarcomeres occurs prior to transition into specific fiber types. This proposed expression pattern particularly holds true for TnI, of which the slow isoform is predominantly expressed in primary and secondary myotubes, and TnC which shows a consistent hybrid expression of fast and slow isoform in myotubes, whereas TnT isoform expression is highly variable (Sutherland et al., 1993). There is significant co-expression of slow and fast isoforms of TnT in developing myotubes prior to the onset of differentiation programming, with significant restriction of isoform gene expression as the myotubes become committed to a fiber type.

### Transcriptional Control of Early Myotube Differentiation

A number of transcription factors, including MyoD, Six, Sox6, Prdm1, Prox1, GTF3, PGC-1ɑ, and Mef2, have been identified with impacts on the differentiation of skeletal muscle fibers. MyoD has been shown to play a significant role in myogenesis, fast skeletal muscle differentiation, and the expression of specific TnT isoforms. Skeletal muscle fibers of MyoD knockout (KO) mice showed significantly greater variability in Ca^2+^-activation as well as greater variability in TnT isoform expression which could affect Ca^2+^ handling, while TnI and TnC isoforms were consistent with those of controls (Metzger et al., 1995; Muroya et al., 2005; Ekmark et al., 2007). The Six family of proteins, especially Six1 and Six4, also upregulate the expression of fast TnT (Niro et al., 2010).

Sox6 gene is another important player in fast skeletal muscle differentiation as Sox6 KO mice show early postnatal lethality coinciding with the formation of secondary myotubes. Sox6 KO results in a decrease in fast skeletal muscle TnI and an increase in slow TnC and slow TnI (Hagiwara et al., 2005). Furthermore, Sox6 acts as a transcriptional repressor of slow-fiber-specific genes in slow skeletal muscle fibers (Hagiwara et al., 2007). Transcription factor Prdm1 is a regulator of slow skeletal muscle, where it counteracts Sox6 repression to encourage slow skeletal differentiation (von Hofsten et al., 2008). Prox1 is another fast skeletal isoform suppressor, and knock-out of the gene increases expression of fast TnT and fast TnI (Petchey et al., 2014). Transcription factor GTF3 binds an upstream enhancer of slow TnI gene, restricting slow TnI expression to slow fibers (Vullhorst and Buonanno, 2003). In addition, PGC-1ɑ overexpression has been shown to drive fast-to-slow fiber-type switching, and PGC-1a is known to interact with Mef2 to upregulate slow TnI and promote slow fiber differentiation (Lin et al., 2002).

### Developmental Regulation of Troponin Isoforms

The three TnT genes show mixed expression in early embryos up until developing neonates, where the expression of fast skeletal, slow skeletal, and cardiac isoforms becomes restricted to their corresponding muscle types as in adults. Studies in mice showed that cardiac TnT is the dominant isoform detected in skeletal muscles of developing embryos and fetuses with co-expression with the embryonic form of MHC. Cardiac TnT is the first detectable TnT isoform in the mesoderm of developing mice at around 8days post coitum (p.c.) while fast TnT and slow TnT are detectable around 12days p.c., coinciding with the decrease in cardiac TnT (Wang et al., 2001). Cardiac TnT is down-regulated in neonates around day 15–20 and ceases expression in adult skeletal muscles (Toyota and Shimada, 1981; Saggin et al., 1990). Alternative splice forms of fast TnT are present at different stages in developing chicken embryo correlating with differences in Ca^2+^ sensitivity of the muscle fibers (Reiser et al., 1992). This was further correlated by the N-terminal acidity of the TnT splice forms, where more acidity confers higher myofilament Ca^2+^ sensitivity of chicken fast skeletal muscle (Ogut et al., 1999).

Troponin I shows significant slow and fast isoform variations during development depending on the species and the muscle group studied. In the biceps femoris of pigs, fast TnI expression was lower in fetus and increased with age while slow TnI expression decreased, corresponding to a decrease in slow MHC I and an increase in fast MHC IIb (Xu et al., 2010). Studies in mice supported this notion and found low but detectable levels of fast TnI in developing embryos which was suppressed over time during postnatal maturation of slow-twitch fibers. Significantly higher levels of fast TnI were found in developing secondary fibers, supporting the hypothesis that secondary fibers largely develop into fast-twitch muscles. This regulation also coincided with the pattern of fast MHC isoforms, suggesting a collectively orchestrated gene regulation that gives rise to fast and slow muscle fibers (Zhu et al., 1995; Hallauer and Hastings, 2002b).

Fast and slow TnI are co-expressed in embryonic muscles, and fast TnI is present from the very beginning of myogenesis. The expression of fast TnI is parallel with fast-twitch MHC IIx in adult mouse muscles (Guerrero et al., 2014). There is a slow-to-cardiac TnI isoform switch during postnatal development of vertebrate hearts where the timing is species dependent based on not birth but functional demands placed on the heart (Hunkeler et al., 1991; Jin, 1996; Krüger et al., 2006). As the functional demands placed on skeletal tissue change dramatically as an organism progresses from embryo to fetus to adult, regulation of fast and slow TnI isoform expressions may also play an adaptive role.

Though studies have indicated that changes in TnC during cardiac development confer unique functions (Posterino et al., 2011), less is known about the developmental regulation of TnC isoforms in skeletal muscles. Fast TnC shows constant expression throughout muscle development while slow/cardiac TnC may show expression in skeletal muscles of early embryos, which becomes undetectable in neonates (Matsuda et al., 1981; Toyota and Shimada, 1981).

## Troponin T Splice Forms are Regulated During Development and in Different Muscles with Functional Significance

### N-Terminal Hypervariable Region

By the mid-1970s, it had become apparent that fast skeletal muscle TnT had different isoforms in chicken leg and breast muscles (Wilkinson, 1978). Monoclonal antibody based studies showed that fast TnT isoforms in these muscles changed during development. All fast muscles of chicken embryos expressed the leg-type isoform. The fast-twitch glycolytic breast muscle expresses the leg isoform early during development before switching to the breast-type within 1week after hatching with some single fibers co-expressing both isoforms (Shimizu and Shimada, 1985; Nakada et al., 2000). Later antibody and molecular cloning studies found that the multiple isoforms of fast TnT are produced by alternative RNA splicing of an N-terminal hypervariable region (Wei and Jin, 2016).

Differential expression of the splice forms of TnT leads to differences in Ca^2+^ sensitivity of tension development, with higher molecular weight isoforms displaying greater sensitivity (Schachat et al., 1987; Greaser et al., 1988; Briggs and Schachat, 1996). Alternative splicing of exons 4–8 results in different contents of acidic residues of the N-terminal variable region that may modulate interactions between TnT and TnC (Briggs and Schachat, 1989). An embryonic-specific exon encodes an N-terminal acidic amino acids-rich segment to produce fetal isoforms significantly more acidic than the adult isoforms, and its inclusion decreases significantly within 7days after birth, regardless of fiber type (Briggs et al., 1990). During vertebrate development, the high-to-low molecular weight and acidic-to-basic isoform switch may function to modulate the tolerance of muscles to acidosis, as the acidic isoforms retain binding affinity for Tm at low pH while Tm-binding affinity of the basic isoforms reduced (Ogut and Jin, 1998).

Slow skeletal troponin T shows an alternative splicing of exons 5 and 6 which also encodes an N-terminal variable region. Two major and one minor slow TnT isoforms are detectable in mouse slow-twitch fibers, and the expression of these isoforms changes with development (Jin et al., 1998a). In contrast to fast TnT, the regulation and functionality are less extensive and no fetal exon is present in the slow TnT gene (Huang et al., 1999b; Yonemura et al., 2000).

The alternative splicing of the three muscle type TnT isoforms is summarized in Table 1 together with the expression of TnC and TnI isoform genes.

**Table 1:** Isoforms and splice forms of troponin subunits.

Isoform gene	Protein product	Expression pattern	Alternative splicing
*TNNC1*	Cardiac/Slow TnC	Cardiac and slow-twitch skeletal muscles	N/A
*TNNC2*	Fast TnC	Fast-twitch skeletal muscle	N/A
*TNNI1*	Slow TnI	Slow-twitch skeletal muscle/Embryonic heart^*^	N/A
*TNNI2*	Fast TnI	Fast-twitch skeletal muscle	N/A
*TNNI3*	Cardiac TnI	Adult cardiac muscle^*^	N/A
*TNNT1*	Slow TnT	Slow-twitch skeletal muscle	N-terminal variable region
*TNNT2*	Cardiac TnT	Cardiac muscle	N-terminal variable region^*^
*TNNT3*	Fast TnT	Fast-twitch skeletal muscle	N- and C-terminal variable regions^*^

The genes encoding vertebrate TnC, TnI and TnT isoforms are listed with their expression in specific muscle types. All three TnT isoforms have evolved with additional diversity *via* alternative splicing. N/A, not applicable.

* Developmentally regulated expression.

### Mutually Exclusive Exons 16 and 17 Encoding a C-Terminal Variable Region of Fast TnT

Different from the slow and cardiac isoforms, the C-terminal region of fast TnT that interacts with TnI and TnC (Wei and Jin, 2016) contains a variable segment encoded by a pair of mutually exclusive exons, 16 and 17 in vertebrates. The C-terminal alternatively spliced variants of fast TnT are the ɑ and β isoforms (Medford et al., 1984). In neonatal skeletal muscles, expression is predominantly exon 17, whereas in adult skeletal muscle expression shows a mixture of both isoforms, except in chicken pectoral muscles where the preference is towards mostly exon 16 (Wang and Jin, 1997; Ogut and Jin, 1998). It is interesting to note that exon 17 shows higher sequence similarity to its counterparts in cardiac and slow TnT compared to exon 16 (Jin et al., 1998b). Within the same fiber, higher levels of the exon 16 product could be detected in the proximal region of 15-day-old chicken gastrocnemius and increased with development (Jozaki et al., 2002). The ɑ splice form of fast TnT produces higher Ca^2+^ sensitivity and ATPase activity in reconstituted myofilaments (Chaudhuri et al., 2005; Gallon et al., 2006).

## Troponin Isoform and Splice Form Regulation and Muscle Usage

### Fatigue Resistance and Exercise

It has been well-established that troponin plays a role in the Ca^2+^-sensitivity of muscle in response to acidosis. Within skeletal muscle cells, the response to acidic pH holds implications for exercise and fatigue resistance, where work-produced local hypoxia and increases in proton production from ATP hydrolysis exceeds the bicarbonate buffering capacity of the tissue (Robergs et al., 2004). Decreases in Ca^2+^-sensitivity following acidosis is most significant in cardiac muscle, followed by fast skeletal and then slow skeletal muscles. While TnC plays the role of Ca^2+^ receptor, slow skeletal and cardiac muscles share the same TnC isoform and thus TnC alone cannot account for the marked difference in acidosis resistance between cardiac and slow skeletal muscle fibers.

*In vivo* studies using reconstituted troponin indicate that TnI-TnC interactions are pH sensitive (Kawashima et al., 1995; Metzger, 1996). Studies in cardiac muscle show that the Ca^2+^ sensitive function of thin filaments decreases at acidic pH, while slow skeletal muscle is more resistant to low pH. Replacing cardiac TnI with slow TnI in skinned cardiac muscle preparations preserves Ca^2+^-sensitivity with decreased pH, implicating slow TnI as a main mediator of the resistance to acidosis in slow skeletal muscle function (El-Saleh and Solaro, 1988; Wattanapermpool et al., 1995).

Studies of muscle fibers expressing TnT isoforms or splice forms showed that more acidity in the N-terminal segment of TnT may also account for acidotic-resistance differences of different skeletal muscles (Ogut and Jin, 1998; Nosek et al., 2004). Altogether, the role of slow isoforms of TnI and TnT in the resistance to acidosis has important functional implications for muscle performance by increasing fatigue resistance.

Endurance training induces marked changes in myosin isoform composition in the muscle. Longitudinal studies showed decreases in type IIb fibers that are offset by increases in type I and type IIa fibers with a corresponding shift to slow forms of troponin, indicating an adaptative shift to more fatigue-resistant fibers (Baumann et al., 1987). On the other hand, athletes trained for muscle power without endurance, such as sprinters or weightlifters, do not show significant muscle fiber conversion and display fast-twitch fatigable fiber composition similar to that of sedentary controls (Jansson et al., 1978).

Studies on rabbit muscle exposed to chronic stimulation mimicking workload showed a fast-to-slow fiber conversion with a switch from MHC IIb to MHC IIa and MHC I (Schachat et al., 1988). Three weeks of chronic stimulation also induced a shift of splice forms of fast skeletal muscle TnT1f and TnT2f to almost entirely TnT3f, which has a lower molecular weight and fewer N-terminal acidic residues (identified by gel mobility and the N-terminal alternative splicing of fast TnT, Table 1). Instead of implicating a decrease in acidosis resistance, the authors hypothesized that TnT3f splice form decreases Ca^2+^-sensitivity as an intermediate in an adaptive shift of fiber types.

Diet plays an important role in muscle fiber type content and growth as well as skeletal muscle glucose homeostasis. One study implicated high-fat diets in conferring a down-regulation of fast TnT and an upregulation in slow TnT in soleus muscle. While no changes were seen in type IIa or IIb fibers, the increase in slow TnT in slow-twitch fibers led to a decrease in force production and relaxation rate, subsequently decreasing exercise potential (Ciapaite et al., 2015).

### Microgravity and Muscle Disuse

Hindlimb unloading in rodents is an effective laboratory approach to alter the mechanical load that skeletal muscles experience. Originally developed to mimic microgravity during spaceflight, by suspending the tail of rats or mice and thus preventing gravity load on hindlimb muscles the impacts of unloading and disuse in conditions such as extended bedrest can be investigated.

In rat and mouse tail suspension models, it is well-established that hindlimb unloading induces a slow-to-fast fiber type transition with increases in the expression of MHC IIx and MHC IIb, opposite to the fast-to-slow fiber type transition seen following endurance training (Stevens et al., 1999). A concurrent transition during hindlimb unloading is a slow-to-fast isoform switch of TnC, TnI, and TnT (predominantly TnT2f that corresponds to higher cooperativity of muscle).

All fiber types in the muscle show atrophy and a reduction in average diameter and normalized tension following hindlimb unloading (Kischel et al., 2001; Bastide et al., 2002; Yu et al., 2007). Unloaded fibers also show a significant decrease in force production and fatigue resistance. Adding reconstituted fast skeletal troponin to unloading-treated fast fibers indicated that not the quantity but instead the isoform types of troponin determine the Ca^2+^ sensitivity of force production (Udaka et al., 2011). The results of these animal models can be extended to humans, where a regimen of resistance and aerobic exercise was shown to significantly prevent the slow-to-fast transition and the accompanying decrease in maximal force in human subjects treated with bedrest (Mounier et al., 2009).

While adaptive changes in the expression of myofilament protein variants begin as early as 3days after unloading (Yu et al., 2007), the recovery after reloading is a much slower process. A study in mice showed that while the expression of MHC I, slow TnI and slow TnT and force production resumed in 2weeks, fatigue resistance did not begin to improve until at least 30days after reloading and remained lower than controls 60days after reloading (Feng et al., 2016). It is worth noting that significant inflammatory responses are seen during the 1st week of reloading after long term unloading, indicating injury due to overloading of the deconditioned and atrophic muscle (Feng et al., 2016).

## Troponin Isoform and Splice Form Regulation in Aging

### Loss of Fast Fibers

Age-related decline in skeletal muscle function results in decreases in muscle mass and force generation together with a loss of motor nerve innervation which is more pronounced in type II fast fibers (Moore et al., 2014). The spatial distribution of motor units becomes more clustered with age, suggesting a pattern of denervation (Wang et al., 2005; Mitchell et al., 2012). As the loss of muscle mass cannot alone account for the loss of force, the loss of motor neuron innervation may have a primary role, particular in the loss of fast fibers. Moreover, proteomics studies reveal changes in myosin light chain 2 corresponding to a fast-to-slow transition of protein isoforms, supporting the notion that aging in muscle shifts the balance towards aerobic-oxidative metabolism in slow twitch fibers (Gannon et al., 2009).

There is evidence to support a role for troponin isoforms in adaptation to aging. Muscles of young and old individuals contain different splice forms of fast TnT. As different TnT splice forms correspond to differences in Ca^2+^ sensitivity, this could affect muscle function in aging. Aging muscle was found to have a decrease in the ɑ splice form relative to the β form of fast TnT. The ɑ splice form is known to impart higher Ca^2+^ sensitivity and ATPase activity, thus its decrease may contribute to the decrease in muscle performance in age (Coble et al., 2015).

Cardiac TnT that is transiently expressed in developing skeletal muscle and following denervation may contribute to the pathophysiology of age-related skeletal muscle decline. It was reported that the levels of cardiac TnT in mouse fast-twitch muscle increased with age and were enriched in the region surrounding the neuromuscular junction in aging mice. Furthermore, knockdown of cardiac TnT in fast-twitch muscle led to an increase in protein kinase A R1ɑ subunit that is known to aid in stability of the postsynaptic neuromuscular junction, suggesting cardiac TnT may act as a regulator of neuromuscular junction function and impact motor nerve functions (Martinez-Pena y Valenzuela et al., 2013; Xu et al., 2017).

### Approaches to Counteract Muscle Loss

While diet is clearly important for skeletal muscle function, the role of glucose metabolism in skeletal muscle aging is multi-faceted and remains incompletely understood. Loss of muscle function not only reduces mobility and function in older individuals, but as skeletal muscle is a major target for glucose uptake, loss of muscle mass may be implicated in the pathogenesis of type 2 diabetes. Exercise training reduces insulin resistance in older adults (Evans et al., 2005; Consitt et al., 2019). In a diabetes-like mouse model of G-protein ɑ subunit deficiency in skeletal muscle with reduced glucose tolerance and low muscle mass, a fast-to-slow fiber type switching occurred upon aging. This phenotype may serve as an adaptive response of aging muscles to functional overload, where the muscle showed better resistance to fatigue despite the overall loss of mass (Feng et al., 2011).

Mounting evidence supports the benefits of exercise intervention in improving the health of aging skeletal muscle. Studies using resistance training have shown improvements in fiber-specific force and power without marked changes in fiber-type composition or muscle cross sectional area (Mitchell et al., 2012; Zhang et al., 2014). An upregulation of the high molecular weight splice form of slow TnT was found in aging individuals who received resistance training over 5months, implicating a role for slow TnT splice forms in muscle adaptation in aging (Zhang et al., 2014). Aerobic training also significantly increases the number of motor units in older individuals, suggesting neuroprotective effects that improve muscle function in old age (Power et al., 2010). However, the benefits of exercise may only be seen after the course of several months (Drummond et al., 2008; Coble et al., 2015).

## Troponin Myopathies and Adaptations in Other Muscle Diseases

As troponin plays a central role in Ca^2+^ regulation of muscle contraction, mutations of troponin subunits have been demonstrated to be involved in the pathologies of some myopathies. Understanding the pathogenesis and pathophysiology of troponin myopathies with specific protein structural abnormalities can provide insights into targeted treatment. The sensitive changes of troponin isoforms and splice forms as a functional adaptation also provide informative markers for the pathophysiology of non-troponin mutation muscle diseases.

### *TNNT1* Myopathies

The most documented troponin-related skeletal myopathies are caused by various mutations in *TNNT1* gene encoding slow skeletal muscle TnT (Mondal and Jin, 2016). The first *TNNT1* myopathy identified was Amish nemaline myopathy (ANM), a lethal recessive nemaline myopathy affecting approximately 1 in 500 births in the Amish communities in Pennsylvania and Ohio (Fox et al., 2018). ANM is caused by a nonsense mutation in exon 11 of *TNNT1* gene resulting in a premature truncation of slow TnT at Glu_180_. Although fast TnT expression remains unchanged, patients with ANM exhibit severe muscle weakness, atrophy of type I fibers, hypotonia and tremors, ultimately resulting in death by age 2–4 from failure of respiratory muscle function. Muscle biopsies from patients with ANM reveal that neither truncated nor intact slow TnT is present, indicating the truncated slow TnT is unable to incorporate into myofilaments (Jin et al., 2003). Normal fetal skeletal muscle expresses predominantly fast TnT and cardiac TnT. When cardiac TnT is down-regulated around birth, slow TnT expression is upregulated. Consistently, patients with ANM present with apparently normal muscle phenotype at birth with myopathy developing postnatally concurrent with the lack of slow TnT expression (Jin et al., 2003; Mondal and Jin, 2016).

A *TNNT1* gene KO mouse model partially reproduced the pathophysiology of ANM. *TNNT1* KO mice do not show premature lethality but have severe type I fiber atrophy and reduction of MHC I, along with decreased force development and resistance to fatigue (Wei et al., 2014). To investigate why the loss of only one isoform of TnT results in severe myopathy despite the abundance of fast TnT, further studies found that slow TnT plays a critical role in the function of the intrafusal fibers of muscle spindles. The loss of slow TnT in intrafusal fibers was partially compensated by an increase in cardiac TnT, resulting in a potential hypersensitivity to Ca^2+^ which may explain the tremors and clonus seen in patients with ANM (Oki et al., 2019).

The characterization of ANM called for *TNNT1* genetic testing in the diagnosis of myopathies, and numerous other mutations have been reported in *TNNT1* gene to cause recessive or conditionally dominant myopathies with progressive muscle deteriorations. Among those reported include a nonsense mutation in exon 9 causing premature truncation at Ser_108_, splicing site mutations causing truncation at Leu_203_ or E_221_, and internal deletion of exon 8, exon 9, or both (Marra et al., 2015; Abdulhaq et al., 2016; Amarasinghe et al., 2016; D’Amico et al., 2019; Géraud et al., 2020). Protein binding studies showed lower Tm binding affinity of Ser_108_ truncated and exon 8 deleted slow TnT. The Leu_203_ truncation mutant retains Tm-binding capacity but lacks TnI and TnC binding sites to also result in complete loss of function (Amarasinghe et al., 2016). Slow TnT mutations that cause deletion of the C-terminal 14 amino acids have been reported to cause ANM-like myopathies in compound heterozygotes with some of the above mutations (van der Pol et al., 2014; Petrucci et al., 2021), confirming the functional importance of the C-terminal end segment of TnT (Lopez Davila et al., 2020).

### Myotonic Dystrophy

Myotonic dystrophies (DM) encompass a group of autosomal dominant inheritance disorders leading to skeletal muscle weakness, wasting, and hyperexcitability, as well as insulin resistance and cardiac dysfunction, affecting approximately 1 in 8,000–20,000 people. The disorder can be traced back to the expansion of CTG repeats in the 3'-untranslated region of the DMPK gene, which encodes for a kinase that is a known regulator of muscle function and may be implicated in many different pathways. A number of genes involved in muscle functions are affected, and among these are *TNNT3* encoding fast TnT and *TNNT2* encoding cardiac TnT. Aberrant splicing of *TNNT3* and *TNNT2* in patients with DM results in altered Ca^2+^-sensitivity of myofilaments (López-Martínez et al., 2020). Differences in fast TnT alternative splicing are seen among patients with distinct forms of DM, indicating *TNNT3* splice forms could be a useful marker for differential diagnosis (Vihola et al., 2010). The splicing abnormality results in shifts to high molecular weight forms of fast TnT. Such splicing shift is also seen in slow TnT, which may also have a causal effect (Salvatori et al., 2009).

### Facioscapulohumeral Muscular Dystrophy

Facioscapulohumeral muscular dystrophy is one of the more common forms of muscular dystrophy, affecting approximately 1 in 15,000–20,000 people worldwide. This autosomal dominant inheritance disorder is characterized by a distinctive muscle weakness and reduced resistance to fatigue in the face, neck, shoulders and upper trunk with onset typically in a patient’s teens or 20s (Tawil, 2018). The disorder occurs as a result of the loss of microsatellite repeats in chromosome 4q35 resulting in hypomethylation of chromatin, with possible targets including genes DUX4 and FRG1. Aberrant slicing of fast TnT has been implicated as a possible cause of the disorder, as FRG1 has been shown to bind to the TnT transcript, and mice overexpressing FRG1 display a shift towards an anomalous acidic fast TnT isoform and an overall switch from a fast-to-slow fiber type. Muscle fibers from FRG1-overexpressing mice show reduced Ca^2+^-sensitive force generation, consistent with the role of fast TnT in modulating myofilament Ca^2+^-sensitivity (Sancisi et al., 2014).

### Distal Arthrogryposis

Distal arthrogryposis (DA) is a rare autosomal dominant disorder affecting joints and reducing function of the distal portions of the limbs without any associated muscle weakness. The disorder is delineated into the more severe form DA1, DA2A (Freeman-Sheldon syndrome), and DA2B (Sheldon-Hall syndrome). Depending on the classification, patients typically present with variable contractures affecting major joints including the hands and feet, while patients with DA2A and DA2B in addition are characterized by facial anomalies including deep-set eyes and small mouth, short stature, and scoliosis. Patients with DA show increased variability of muscle fiber size, largely confined to type II fibers. In some patients with DA, an in-frame internal deletion in *TNNI2* gene results in a deletion of Lys_176_ from fast TnI. Lys_176_ is a highly conserved residue and mutation of the corresponding residue in cardiac TnI causes hypertrophic cardiomyopathy. Loss of this Lys in cardiac TnI results in an increase in Ca^2+^ sensitivity and a similar mechanism may be at play in DA skeletal muscles. Patients with DA with mutations of the *TNNT3* gene that alter Ca^2+^ sensitivity may have a similar mechanism of pathogenesis (Kimber et al., 2006; Toydemir and Bamshad, 2009), and *TNNT3* mutations may lead concomitantly to DA and nemaline myopathy of fast twitch fibers (Sandaradura et al., 2018).

### Adaptive Changes of Troponin in Other Myopathies

Charcot–Marie–Tooth disease (CMT) is a hereditary disorder affecting the peripheral nerves. CMT type 1 is classified by peripheral axon demyelination, resulting in reduced motor nerve conductive velocity, while CMT type 2 is classified by axonal degeneration. Patients with both CMT type 1 and type 2 experience weakening of muscles in the periphery limbs, including weakness and muscle atrophy of the feet and hands (Miniou and Fontes, 2021). In biopsies of patients with CMT type 1 slow fibers showed a significant up-regulation of the low molecular weight splice form of slow TnT, while no changes in troponin were observed in patients with CMT type 2. This difference may suggest that the change in the quality of neuronal inputs, such as that from axon demyelination in CMT type 1 vs. that from axon loss in CMT type 2, triggers an adaptation in sarcomeric contractile apparatus *via* alternative splicing of TnT to alter the thin filament regulation. The increase in low molecular splice forms of slow TnT in CMT type 1 muscle may have the effect of increasing force production as the less acidic slow TnT splice form confers increased Ca^2+^-activated tension (Larsson et al., 2008).

Abnormal splicing regulation of the mutually exclusive exons 16 and 17 of fast TnT gene *TNNT3* was found in oculopharyngeal muscular dystrophy, resulting in changes in Ca^2+^ sensitivity (Klein et al., 2016). Biallelic *TNNT3* mutations were further found to be associated with a severe recessive congenital myopathy in patients presenting with or without nemaline rods and DA (Calame et al., 2021).

There are recently reported cases of TnC causing skeletal muscle myopathies. Inherited fast TnC (*TNNC2*) missense mutations led to scoliosis and respiratory weakness at birth among members of two families studied, though the condition appears to improve over time and suggests a compensatory effect of cardiac/slow TnC with increased reliance on slow twitch fibers with age. Unlike mutations in slow TnT, the reported TnC mutations do not appear to cause nemaline rods or dramatically alter the myofibrillar structure and solely act through decreasing Ca^2+^-sensitive force generation of the fibers (van de Locht et al., 2021).

In contrast, some myopathies, such as TP3 myopathy, acute quadriplegic myopathy, and neurogenic muscle atrophy, show changes in troponin subunits corresponding to atrophy of slow and/or fast fibers but appear to display no unique or abnormal loss or alteration of troponin (Furukawa and Peter, 1972; Matsumoto et al., 2000; Yuen et al., 2015).

### Other Applications of Troponin Biomarker for the Treatment of Human Diseases

Muscle regeneration, either through grafts, stem cells, or satellite cells, has been a key goal for improving human health. As technologies develop, tissue engineering will continue to be a key approach in researchers’ toolbox, though difficulties with innervation, vascularization, and inflammatory reactions remain (Liu et al., 2018; Alarcin et al., 2021). Engineered tissues are also a means to study muscle function *in vitro* without the need of biopsies and open the possibility to induce disease-related proteins to study function (Abdul-Hussein et al., 2012; Khodabukus and Baar, 2015). Myofilament protein expression is directly informative to delineating myogenesis and muscle tissue engineering as it reflects contractile functions. Expression of adult forms of troponin subunits represent key markers for the differentiation of stem cells into functioning myocytes and the maturity of regenerated or engineered muscles (Wheelwright et al., 2020).

## Conclusion

Myofilament Ca^2+^-regulation is a key determinant of skeletal muscle function, and troponin is the crucial regulator of Ca^2+^-dependent contraction and relaxation. While the expression pattern of MHC is complex and often involves co-expression of multiple isoforms within the same fiber, most fibers express only one isoform of TnI, TnT, and TnC. As fast and slow skeletal fibers differ markedly in their sensitivity to Ca^2+^, troponin plays a key role in defining fiber-specific functions. Thus, troponin isoforms may present a specific marker for classification of fast and slow skeletal fiber types as well as evaluation of developmental and differentiation states.

The importance of delineating fiber types can be seen in a number of muscle wasting and weakness disorders such as ANM and DM, where troponin mutations or dysfunctional aberrant splicing acts with precision on one specific fiber type. With data from models of age-related muscle decline, disuse, or exercise training, we are presented with a means to understand changes in troponin isoform expression for use in positively impacting human health by improving the Ca^2+^-handling ability and specific force generation of skeletal muscles. A clear understanding of protein structure–function relationship informs how changes at the myofilament level confer functions at the fiber level, and the troponin subunit proteins present attractive markers for understanding muscle fiber-type-specific functions, growth and regeneration, age-related decline, myopathies, and compensatory adaptations.

## Author Contributions

J-PJ conceived the topic of research. MR drafted the manuscript. MR and J-PJ edited and revised the writing. All authors contributed to the article and approved the submitted version.

## Funding

This work was supported in part by grants from the National Institutes of Health HL127691 and HL138007 to J-PJ.

## Conflict of Interest

The authors declare that the research was conducted in the absence of any commercial or financial relationships that could be construed as a potential conflict of interest.

## Publisher’s Note

All claims expressed in this article are solely those of the authors and do not necessarily represent those of their affiliated organizations, or those of the publisher, the editors and the reviewers. Any product that may be evaluated in this article, or claim that may be made by its manufacturer, is not guaranteed or endorsed by the publisher.
